# Neurotoxic Myelitis Following Accidental Epidural Injection of Chlorhexidine During Obstetric Epidural Anesthesia: A Case Report

**DOI:** 10.7759/cureus.84299

**Published:** 2025-05-17

**Authors:** Badie Douqchi, Omar Alaoui Mhammedi, Doaae El Ouaddane, Amine Elmouhib, Mohammed El Aissaouy, Houssam Bkiyar, Brahim Housni

**Affiliations:** 1 Department of Anesthesia and Reanimation, Mohammed VI University Hospital, Faculty of Medicine and Pharmacy, Mohammed First University, Oujda, MAR

**Keywords:** anesthesia, case report, chlorhexidine myelitis, intrathecal injection, neurotoxic myelitis

## Abstract

We report a case of neurotoxic myelitis following the accidental epidural injection of chlorhexidine during obstetric anesthesia at a peripheral hospital. A 32-year-old parturient in labor received an epidural catheter for vaginal delivery, during which 3 mL of chlorhexidine was mistakenly injected into the epidural space. This led to progressive paraplegia, severe headaches, and respiratory distress requiring intubation. The patient was subsequently transferred to our anesthesia and critical care department for further management. Radiological findings revealed centromedullary edema and myelitis. Her condition improved with steroid therapy to reduce the inflammatory response, along with strict monitoring. Paraplegia nearly resolved within 72 hours, with complete functional recovery one week later. This study highlights the serious risks of accidental intrathecal drug administration and underscores the need for heightened vigilance and safety measures in anesthesia practice.

## Introduction

The accidental administration of the wrong drug to patients is a well-known issue in clinical practice. This unintentional act is most commonly observed in intravenous drug administration, and rarely in intrathecal injections. Although there have been isolated reports linking chlorhexidine to neurotoxicity, no clinical evidence in humans supports this association [1]. Chlorhexidine-related neurotoxicity would likely manifest as arachnoiditis or aseptic meningitis. Unfortunately, there is no specific clinical guideline for managing such events [2]. This study describes the accidental epidural injection of chlorhexidine, resulting in spinal cord lesions due to the neurotoxic effects of the substance. The management strategy centered on strict monitoring of the patient's sensory and motor functions. This study aimed to raise awareness of the serious complications that can arise from such inadvertent errors.

## Case presentation

A 32-year-old parturient at full term in labor was scheduled for epidural analgesia in preparation for vaginal delivery at a peripheral hospital. During epidural catheter placement, 3 mL of chlorhexidine was inadvertently injected into the epidural space. The patient rapidly developed progressive paraplegia and was subsequently transferred to our intensive care unit for further management. Upon admission, she exhibited severely reduced muscle strength, graded 1/5 in both lower limbs, along with paresthesia in both upper limbs and severe, persistent headaches. Acute respiratory distress ensued, necessitating emergency orotracheal intubation. A comprehensive panel of laboratory tests was conducted upon admission, with the results shown in Table 1.

**Table 1:** Admission laboratory results.

Parameters	Obtained value	Reference range
White blood cell count	9x10^9^/L	4.0-10.0x10^9^/L
Hemoglobin	13 g/dL	12.0-15 g/dL
Platelet count	225x10^9^/L	150-400x10^9^/L
C-reactive protein (CRP)	11 mg/L	<5 mg/L
Lactate	3.2 mmol/L	0.5-2.2 mmol/L
Arterial blood pH	7.33	7.35-7.45
PaO_2_	75 mmHg	80-100 mmHg
PaCO_2_	38 mmHg	35-45 mmHg
Serum creatinine	1 mg/dL	0.6-1.2 mg/dL
Serum sodium	138 mmol/L	135-145 mmol/L
Serum potassium	4.5 mmol/L	3.5-5.0 mmol/L

**Figure 1:**
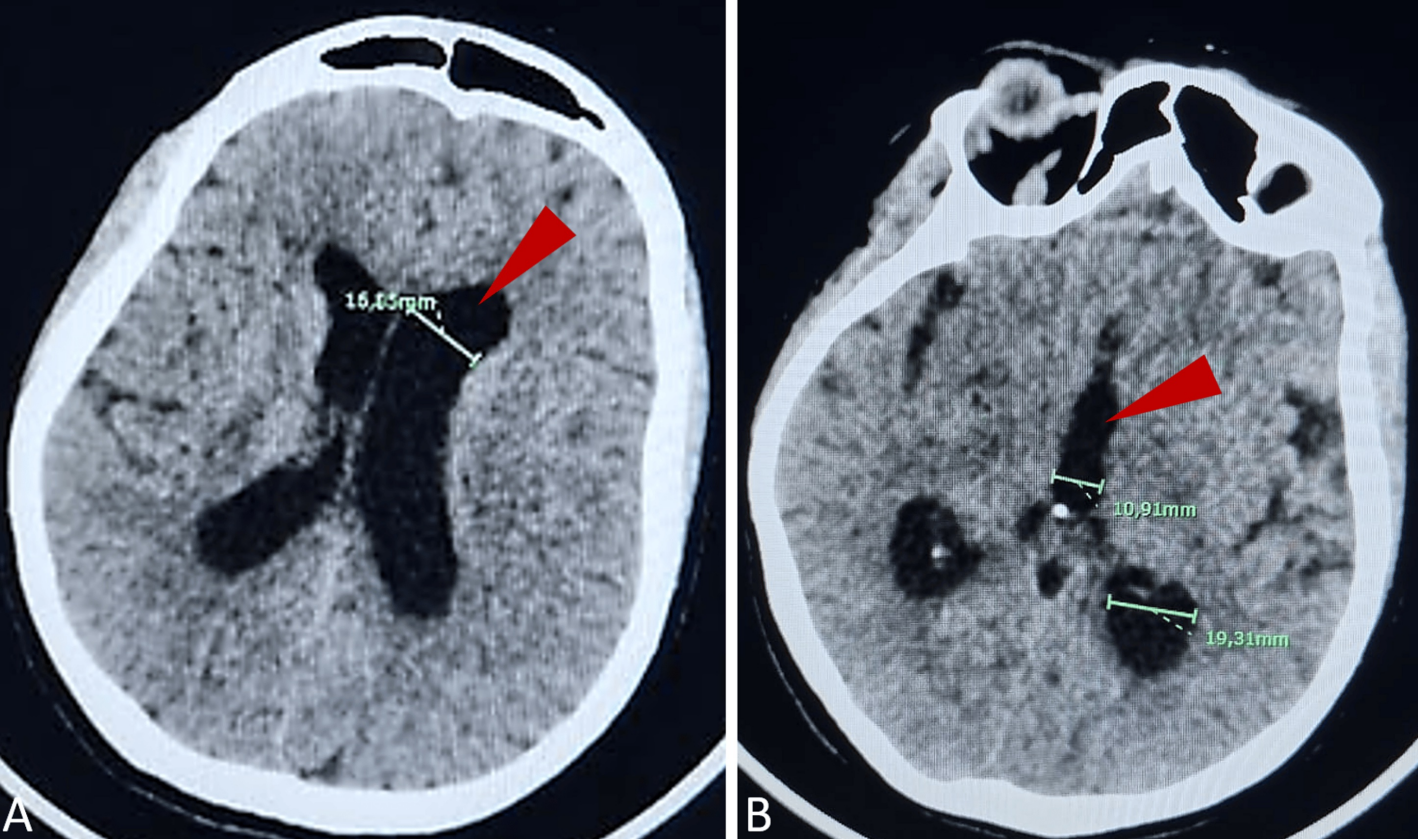
Transverse CT section of the brain showing hydrocephalus (arrow head) of the lateral ventricles (A) and the third ventricle (B).

**Figure 2:**
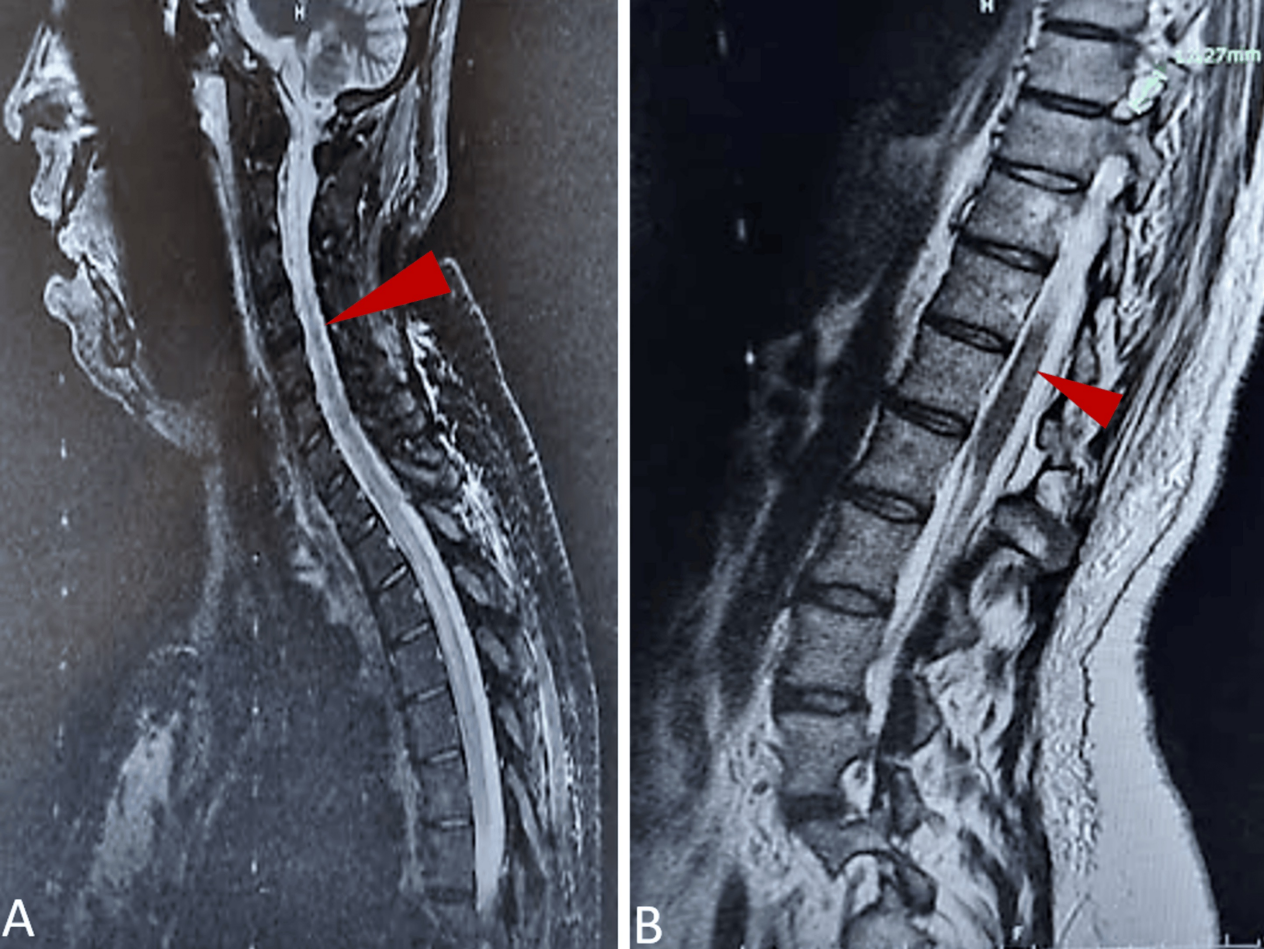
Sagittal section MRI of the spine showing centromedullary edema in the lower cervical region (arrow head) (A). Sagittal section MRI of the spine showing centromedullary edema extending to the conus medullaris (arrow head) (B).

Radiological investigations were conducted once the patient was stabilized, including a brain CT and contrast-enhanced spinal MRI. The non-contrast brain CT showed hydrocephalus in the lateral ventricles (Figures 1A, 1B). The spinal MRI revealed continuous centromedullary edema extending from the lower cervical region to the conus medullaris (Figure 2A), with myelitis and suspected ischemia of the thoracolumbar segment (Figure 2B).

The patient was initially treated with a corticosteroid bolus (methylprednisolone 1 g IV over three days) and was placed on mechanical ventilation, although there were difficulties in weaning her off the ventilator. Sensory disturbances in the upper limbs resolved within the first 48 hours, while paraplegia persisted, graded at 2/5 in the lower limbs. Clinical evaluation 72 hours post-injection revealed mild residual paraplegia, with muscle strength graded 1/5 in both lower limbs and normal neuromuscular function in the upper limbs. Sensory examination revealed mild paresthesia in both upper limbs, with partial loss of sensation below the T10 in the lower limbs. Deep tendon reflexes were markedly diminished in the lower limbs but were exaggerated in the upper limbs. Cranial nerve examination showed no obvious facial weakness or other significant deficits. Supportive care included fluid resuscitation, hemodynamic stabilization, and physical therapy. The patient gradually regained motor function, achieving complete resolution of paraplegia one week after admission. There were no residual headaches or long-term neurological deficits upon discharge.

## Discussion

Intrathecal regional spinal anesthesia, commonly used in obstetric anesthesia, often involves a bolus injection of local anesthetics for a dense sensory-motor block [3]. Chlorhexidine, a potent and broad-spectrum germicide, is effective against most nosocomial yeasts, Gram-positive and Gram-negative bacteria, and certain viruses [4]. The effects of chlorhexidine on neural tissues, such as the spinal cord and meninges, are poorly documented due to the rarity of such incidents. However, animal studies have shown profound histological and clinical evidence of neurotoxicity when high concentrations of chlorhexidine are applied to the middle ear, anterior chamber of the eye, or directly to the neuraxis [5]. Other studies have also demonstrated neurotoxicity when chlorhexidine is applied to Schwann and neuroblastoma cells [6].

Due to the lack of clinical data and early laboratory studies that suggest a link between chlorhexidine and neurotoxicity, the US Food and Drug Administration warns against using chlorhexidine for lumbar punctures or direct contact with the meninges [1]. The concern about neurotoxic antiseptic agents used prior to spinal surgeries is limited to open spinal wounds, a scenario for which data on human subjects is scarce [6]. Neurotoxicity from intrathecal injections has been mostly associated with anesthetic agents as a rare side effect. Accidental intrathecal injection of neurotoxic substances like chlorhexidine is highly unusual, with only a few other cases reported in the literature [7]. A similar incident, in which 8 mL of chlorhexidine was mistakenly injected epidurally, resulted in paralysis and required neurosurgical intervention [8].

In cases of immediate inflammatory responses to intrathecal anesthetic administration, clinical manifestations can include hydrocephalus, cervicothoracic syringomyelia, arachnoiditis with cauda equina dysfunction, and ultimately paraplegia and motor function loss [9]. Myelitis from post-intrathecal injection may also result from chlorhexidine contamination during skin preparation, which acts as the inflammatory stimulus [10].

Management of such cases requires strict monitoring of the patient's clinical condition. Some studies have suggested cerebrospinal fluid (CSF) lavage, involving direct aspiration and replacement of CSF, to prevent meningitis or encephalitis. However, more research is needed to confirm the efficacy of this technique [11]. The management of inadvertent intrathecal vincristine injections, which has been extensively reviewed, suggests that CSF lavage is the most common intervention based on available literature. In contrast, simple CSF aspiration is insufficient, as it removes only minimal amounts of the drug and has been associated with poor outcomes [12]. Prolonged CSF irrigation, maintained for over 24 hours and combined with continuous drainage via lumbar and ventricular catheters, has been shown to improve survival and limit neurological damage. Neuroprotective agents such as pyridoxine, folic acid, and corticosteroids may help prevent further neuronal damage [13].

To reduce medication administration errors during anesthesia in the operating room, the European guidelines require the use of standardized labeling practices, including color-coded and clearly marked syringes, to prevent medication errors. The use of pre-filled and pre-labeled syringes, especially for high-risk drugs, is encouraged to minimize dilution errors and contamination. Strict double-checking protocols and barcode scanning systems are recommended to verify medications [14].

In the United Kingdom, the Guidelines for the Provision of Anaesthetic Services (GPAS) encourage anesthetists to prepare and label their own medications, ideally using pre-filled syringes with standardized labeling compliant with ISO 26825. Minimizing the number of syringes drawn up, ensuring proper storage, and maintaining timely access to emergency medications are also critical. Medication errors are often linked to look-alike drugs, unclear labeling, or changes in drug presentation issues that can be mitigated through robust communication, standardized packaging, and pharmacist oversight. These recommendations align with the broader national objective of reducing medication-related harm, in line with the WHO's Global Patient Safety Challenge [15].

NRFit syringes and connectors, specifically designed for neuraxial and regional anesthesia, aim to prevent wrong-route medication errors. Their distinct yellow color and unique connector design render them incompatible with standard Luer systems, thereby eliminating the risk of accidental intravenous administration of neuraxial drugs. The system enhances patient safety, requires minimal training, and represents a cost-effective safety upgrade for operating room practices [16]. Although NRFit-compatible syringes are not yet available in our hospital, their introduction in the operating room could significantly reduce the risk of such errors.

According to the American Society of Health-System Pharmacists (ASHP) recommendations, preventing accidental drug administration requires clear labelling, segregation of medications, independent double-check procedures, and closed-loop communication. Promoting a culture of error reporting and applying proactive risk assessment tools are also essential to reducing the risk of medication errors [17].

In the operating room, we use sterile, disposable, color-coded medication cups that are filled from properly labeled ampoules. However, a critical error occurred when a physician confused two cups containing clear liquids, normal saline, and chlorhexidine, due to the absence of proper labeling on the cups. This mistake happened during syringe preparation and highlights the need for reliable identification systems to prevent medication errors. In the present case, strict monitoring of the patient's sensory and motor functions, along with corticosteroid administration, was carried out due to the lack of a well-established management protocol. Remarkably, the patient's motor function gradually improved, and paraplegia completely resolved 72 hours after the intrathecal chlorhexidine injection.

## Conclusions

This case highlights a severe and potentially life-threatening complication following inadvertent chlorhexidine injection into the epidural space. The high-risk nature of the delivery room environment necessitates stringent protocols to minimize medication errors. The present case emphasizes the critical need for standardized prevention measures, early recognition, and a multidisciplinary management approach in such scenarios. Additionally, this case underscores the importance of rapid intervention, including corticosteroid therapy and supportive care, to improve neurological recovery. Key lessons learnt include the necessity of clearly identifying drugs through appropriate labelling and storage, ensuring effective team communication, and conducting regular training and safety drills to reinforce protocol adherence. Future strategies should focus on increasing awareness, implementing clear labeling and storage protocols, and reinforcing training among healthcare professionals to prevent similar occurrences.
